# Fine needle Aspiration Biopsy (FNAB) in the initial evaluation and diagnosis of palpable soft tissue lesions and with histologic correlation

**DOI:** 10.11604/pamj.2015.20.44.4271

**Published:** 2015-01-15

**Authors:** Gabriel Olabiyi Ogun

**Affiliations:** 1 Department of Pathology, College of Medicine, University of Ibadan/ University College Hospital, Ibadan, Nigeria

**Keywords:** FNAB, soft tissue lesion, histology, correlation

## Abstract

**Introduction:**

Fine-needle aspiration biopsy (FNAB) as a means of evaluation of palpable soft tissue lesions is poorly utilized in our environment despite the fact that it safe, cheap, quick and easy to perform.

**Methods:**

All cases of cases of palpable soft tissue lesions of the trunk and extremities where FNAB was used as the initial evaluation tool were reviewed. Furthermore, the records for corresponding cases that had open excision biopsy and ultimately had histologic diagnosis out of these cases were also retrieved and correlated with the final diagnosis from FNAB.

**Results:**

Out of 142 aspirates, only 107(75.3% of cases) fulfilled the inclusion criteria for the study. The age range was from 0-85 years (mean = 41.2 yrs.) with a roughly equal male:female ratio. The lesions were located in the trunk -56 cases, upper arm -7, forearm -1, hand -1, thigh -28, leg -7 and the foot-7. The FNAB was diagnosed as benign in 56 (52.3%) cases, malignant in 48 (44.8%) cases and suspicious of malignancy in 3(2.8%) cases. The cases were cytomorphologically classified into the following categories: Lipomatous (32 cases), epithelia (18), spindle cell (14), inflammatory (13) pleomorphic (11), small round (6), myxoid (5), epitheloid/ polygonal (1) and others (7). The sensitivity and specificity of diagnosed cases with FNAB as either benign or malignant when correlated with histology were 95% and 100% respectively.

**Conclusion:**

FNAB is a valuable tool in the initial evaluation of palpable soft tissue lesions especially in primary soft tissue neoplasms and clinically suspected metastatic carcinomas.

## Introduction

Palpable soft tissue masses are quite common in clinical practice. The use of Fine needle aspiration biopsy (FNAB) remains highly underutilized in our environment despite the fact that it is safe, cheap, quickly, easy to perform and not usually fraught with complications [1, 2]. The FNAB is handy as a means of early diagnosis and help prevent unnecessary and expensive open biopsies [3, 4]. With increasing knowledge, experience and training in the use of FNAB in the diagnosis of soft tissue lesions and palpable lumps in other parts of the body, it is increasingly becoming common place in the practice of cytopathology in some sub-Saharan Africa countries and globally [5–7]. Many reports from different parts of the world suggest that the FNAB has high diagnostic accuracy and value in experienced hands [1, 7–9]. The issue of cost is more important and significant in sub-Saharan and developing African countries where there are many competing needs for limited funds allocated to health care on the part of government and also from patients who pay for health care out-of-pocket [3, 10]. The aim of this study was to assess the diagnostic efficacy of the use of FNAB as an initial assessment tool for palpable soft tissue lumps and swelling in the trunk and extremities, determine the accuracy of FNAB and correlate the final cytologic diagnosis with the histopathologic diagnosis in patients that ultimately had open tissue biopsy performed.

## Methods

The records of consecutive palpable soft tissue lesions of the trunk and extremities that had FNAB performed as at the fine needle aspiration cytology clinic of the Department of Pathology University College Hospital were reviewed for a period 5 years. All cases included for analysis had adequate smears in terms of material available on the slides for assessment. Lesions in the head and neck, the breast, lymph nodes, intraabdominal and intrathoracic locations were excluded from the study. Only cases with sufficient clinical information and a definitive final cytologic diagnosis were included in the study. Furthermore, records for corresponding cases that had open excision biopsy out of these cases were also retrieved. All the cytology smears and histology were reviewed by the author to ascertain the correctness of diagnosis.

A registered nurse was always in attendance during the procedure and served as chaperone. The cytology material is usually obtained by conventional method of fine needle aspiration using either a 21 or 23 gauge needleattached to a 10ml disposable plastic syringe. Negative pressure is created by pulling the plunger once the needle is inserted into the nodule or mass and firm suction applied with short strokes in different directions in the lesion depending on the size of the lesion. Material obtained by the aspiration is expelled onto glass slides and smears made with another glass slide on a minimum of three slides, two of which were fixed immediately in 95% alcohol for a minimum of 30 minutes and one air dried. In some cases more than three slides were made for each aspiration. The immediately fixed slides are routinely stained with Papanicolaou staining method while the air dried slides are stained with May Grunwald-Giemsa (MGG) stain and occasionally with haematoxylin and eosin (H&E) stains. Sometimes, the FNAB is repeated if the material obtained at the first aspirate is not adequate for cytologic assessment. All adequate smears were group into three broad categories - benign, malignant and suspicious of malignancy. Furthermore all adequate smears were reclassified during the review by the author based on cytomorphologic features into lipomatous, epithelia, spindle cell, inflammatory, pleomorphic, small round cell, myxoid, epitheloid/polygonal and other lesions [1]. Also anatomic location of the lesions were stratified into the trunk, upper limb (upper arm, fore arm and hand) and lower limb (thigh, leg and foot). This study was conducted in compliance with the guidelines of the Helsinki declaration on biomedical research in human subjects. Confidentiality of the identity of the patients and personal health information was maintained.

## Results

**Figure 1:**
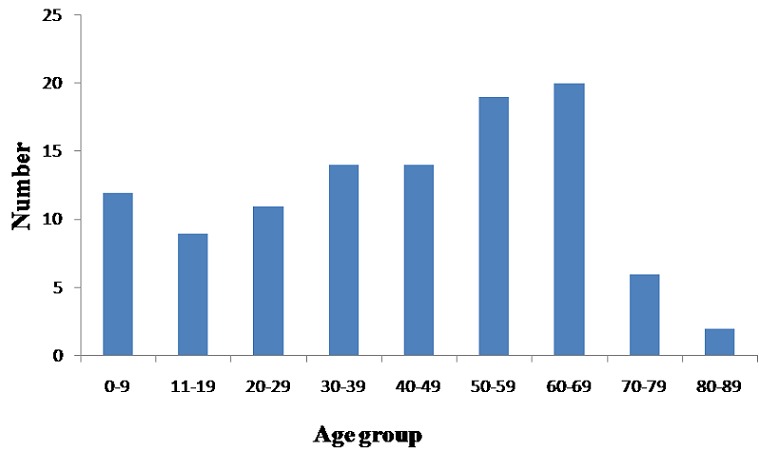
Age group distribution of patients that had FNAB as initial evaluation of soft tissue lesion of the trunk and extremities

There were a total of 142 soft tissue lesions assessed for this study from 141 patients.(A patient had two lesions). However only 107(75.3% of cases assessed) palpable soft tissue lesions in 107 patients fulfilled the inclusion criteria and were suitable for analysis. The rest were either inadequate for diagnosis (12%) or did not have enough clinical information. There were 56 males and 51 females giving a roughly equal male: female ratio. The age range for the patients was from 0 - 85years with a mean age of 41.2 years. Figure 1 show the age group distribution of the patients. The lesions were located in the trunk -56 cases, upper arm -7, forearm -1, hand -1, thigh -28, leg -7 and the foot-7. The final cytologic diagnosis was benign in 56 (52.3%) cases, malignant in 48 (44.8%) cases and suspicious of malignancy in 3(2.8%) cases. Table 1 shows the cytomorphologic classification of the final cytologic diagnosis into benign, malignant and suspicious of malignancy. From Table 1, of the 18 cases are classified as epithelia lesions, 16 of which were diagnosed as metastatic carcinomas. Only 40 (37.8%) of cases that had FNAB as the initial evaluation of palpable soft tissue lesion had open biopsy as a management modality from our records. Hence they had a histologic diagnosis. Table 2 show details of cases that had histology done and the final histologic diagnosis. The correlation tests between the broad final cytologic diagnosis (benign, malignant and suspicious of malignancy) and the histologic diagnosis in those that had open biopsy are as follow- there were 19 true positives (FNAB malignant and histologic diagnosis malignant) and 20 true negatives (FNAB benign and histologic diagnosis benign). There was no false positive case (FNAB malignant and histologic diagnosis benign) and 1 false negative case (FNAB benign and histologic diagnosis malignant) thereby giving a false negative rate of 2.5%. The sensitivity and specificity of diagnosed palpable soft tissue lesions of the trunk and extremities with FNAB across the Benign and Malignant classification, with histology as the gold standard for diagnosis were 95% and 100% respectively. The positive predictive value (PPV and negative predictive value (NPV) were 100% and 95% respectively. The overall accuracy of FNAB with histologic correlation was 97.5%.

**Table 1:** Cytomorphologic classification of the final cytologic diagnosis of 107 cases

	Cytomorphology	Benign	Malignant	Suspicious	Total
1	Lipomatous	32	-	-	32
2	Epithelia	-	18	-	18
3	Spindle cell	4	9	1	14
4	Inflammatory	13	-	-	13
5	Pleomorphic	-	11	-	11
6	Small round cell	-	6	-	6
7	Myxoid	3	2	-	5
8	Epitheloid/Polygonal	1	-	-	1
9	Others	3	2	2	6
	Total	**56**	**48**	**3**	**107**

**Table 2:** Details of cases that had histology done and with the final histologic diagnosis

Cytomorphologic classification (1)	Benign, Malignant or suspicious of malignancy by FNAB diagnosis	Number that had open biopsy and Histology	Final Histologic diagnosis and Number for each diagnosis
Lipomatous	Benign	16	Lipoma (13 cases) Condyloma (1) Fibromatosis (1) Haemangioma(1)
Spindle cell	Benign	1	Dermatofibroma
	Malignant	5	MPNST (2 cases) MFH (2) Fibrosarcoma (1)
Pleomorphic	Malignant	8	Aggressive haemangiopericytoma PRMS, Met PD HCC, Met PD IDC, HG MPNST, Chondrosarcoma. Pleomorphic MFH(2cases)
Myxoid	Benign	2	Osteochondroma, Met Mucinous Adenocarcinoma*
Epithelia	Malignant	4	SCC (2 cases -1 primary, 1- Met); MM; Met Carcinoma from stomach
Inflammatory	Benign	2	Idiopathic tumoral calcinosis with abscess, Wall of abscess cavity
Epitheloid/Polygonal	Suspicious of malignancy	1	Epitheloid haemangioendothelioma
Others (Reported as malignant connective tissue tumour)	Malignant	1	Synovial sarcoma

MPNST- Malignant Peripheral Nerve Sheath Tumour, MFH- Malignant Fibrous Histiocytoma, PRMS- Pleomorphic Rhabdomyosarcoma, Met- Metastatic, PD- Poorly Differentiated,HCC- Hepatocellular Carcinoma, IDC- Invasive Ductal Carcinoma, HG - High Grade, SCC- Squamous Cell Carcinoma, MM- Malignant Melanoma *FNAB reported as benign but Malignant on histology (the only False negative case)

## Discussion

This study illustrates the high efficacy of the FNAB in discriminating between benign and malignant palpable soft tissue lesions. An accuracy of 97.5% when a correlation with open biopsy for the same cases is performed based on histologic diagnosis. This is quite critical and important in streamlining patients’ management and making clinical decisions. However, the limitation of the FNAB is obvious because of the varied lesions in Table 2 that can present as palpable soft tissue lesions, which include primary and secondary neoplastic lesions, inflammatory and reactive lesions as indicated from the histologic diagnosis. This study illustrates that FNAB can be used in all age group of patients. However, it was disappointing that only 40 cases representing 37.8% of patients with FNAB had a histology diagnosis we could compare with. This relatively low number of excision biopsy may be due to a number of reasons including - patient not having excision biopsy at all, FNAB diagnosis and clinical course with radiologic follow up was satisfactorily benign and patients completely lost to follow up or died before open biopsy. This study indicated that 12% of the cases were inadequate for cytologic diagnosis; this is similar to 17% reported by Layfield et al [11]. The reasons that might account for inadequate aspirates include poor technique, necrosis or cystic change in malignant lesions; and in lesions like desmoids and keloids which are fibroblastic and aspiration of material for cytolopathologic diagnosis is difficult from such lesions. [11–13].

In this study the sensitivity and specificity of FNAB in stratifying soft tissue lesions into benign and malignant lesions were 95% and 100% respectively. There was a single case of false negativity giving a 2.5% rate and no false positive case. Wakely and Kneisl in a series of 82 cases of soft tissue lesions analysed by FNAB reported a sensitivity and specificity of 100% and 97% respectively and a single false negative case; a result which is similar to that of the current study [12]. Also, Layfield et al and Nagira et al in their studies of FNAB of soft tissue lesions, reported sensitivity and specificity that range from 92% to 97% [1, 11]. This current study show similar findings to that of Kilpatrick et al, they reported one false negative case and no false- positive case in a study of 145 consecutive sarcomas of bone and soft tissue while Akerman and Rydholm in a study of 517 soft tissue tumors reported a false negative rate of 2.9% and false positive rates of 2.9% [6, 14]. The positive and negative predictive value of 100% and 95% in this study is similar to those of Nagira et al who recorded 93% and 96% respectively [1].

The results in Table 2 illustrate the obvious superiority of Open biopsy with subsequent histologic diagnosis after an initial FNAB assessment. Based on cytomorphology, this superiority is most obvious considering the pleomorphic lesions. These lesions are varied and with widely different histogenesis of the tumours. Our findings are similar to those previously observed and reported [1, 15–17]. Nagira et al and Klijanienko et al reported only 24.2% and 28% of cases of Pleomorphic lesions were rendered precisely by FNAB when correlated with histologic diagnosis [1, 17]. In the current study, all epithelia lesions especially metastatic carcinomas were precisely diagnosed on FNAB when correlated with histology. This is easily attainable in cytopathology practice, when the diagnosis is correlated with clinical history of the patient. The precise FNAB diagnosis in lipomatous lesions was correct in 13 of the 16 cases in the present series. All the 13 cases were lipomas. In this study there was no record of liposarcomas on histology to correlate with FNAB. The findings regarding FNAB and histologic correlation in lipomas are similar to those of Nagira et al [1]. In summary FNAB smears of 107 cases and 40 corresponding histologic cases were examined. The sensitivity and specificity of FNAB across the benign and malignant stratification was 95% and 100% respectively and with a PPV and NPV of 100% and 95% respectively.

## Conclusion

FNAB is a valuable tool in clinical medical practice in stratifying palpable soft tissue lesions into benign and malignant categories and it can be used in the initial evaluation of all palpable soft tissue lesions especially in primary soft tissue neoplasms and clinically suspected metastatic carcinomas.
